# An intercomparison of the pore network to the Navier–Stokes modeling approach applied for saturated conductivity estimation from X-ray CT images

**DOI:** 10.1038/s41598-021-85325-z

**Published:** 2021-03-12

**Authors:** Bartłomiej Gackiewicz, Krzysztof Lamorski, Cezary Sławiński, Shao-Yiu Hsu, Liang-Cheng Chang

**Affiliations:** 1 Institute of Agrophysics, Polish Academy of Sciences, Doświadczalna 4, 20-290 Lublin, Poland; 2 Department of Bioenvironmental Systems Engineering, National Taiwan University, Taipei, 10617 Taiwan; 3 Department of Civil Engineering, National Chiao Tung University, 1001 University Road, Hsinchu, 30010 Taiwan

**Keywords:** Hydrology, Computational science

## Abstract

Different modeling techniques can be used to estimate the saturated conductivity of a porous medium based on computed tomography (CT) images. In this research, two methods are intercompared: direct modeling using the Navier–Stokes (NS) approach and simplified geometry pore network (PN) modeling. Both modeling approaches rely on pore media geometry which was determined using an X-ray CT scans with voxel size 2 μm. An estimate of the saturated conductivity using both methods was calculated for 20 samples prepared from sand with diverse particle size distributions. PN-estimated saturated conductivity was found to be statistically equivalent to the NS-determined saturated conductivity values. The average value of the ratio of the PN-determined conductivity to the NS-determined conductivity (K_satPN/NS_) was equal to 0.927. In addition to the NS and PN modeling approaches, a simple Kozeny-Carman (KC) equation-based estimate was made. The comparison showed that the KC estimate overestimated saturated conductivity by more than double (2.624) the NS estimate. A relationship was observed between the porous media specific surface and the K_satPN/NS_ ratio. The tortuosity of analyzed samples was estimated, the correlation between the porous media tortuosity and the specific surface of the samples was observed. In case of NS modelling approach the difference between pore media total porosity and total porosity of meshes, which were lower, generated for simulations were observed. The average value of the differences between them was 0.01. The method of NS saturated conductivity error estimation related to pore media porosity underestimation by numerical meshes was proposed. The error was on the average 10% for analyzed samples. The minimum value of the error was 4.6% and maximum 19%.

## Introduction

Pore-scale models of flow and transport in porous media have various applications in geophysics, engineering or environmental modeling. These models can be divided into two general groups: pore network modeling (PNM), which uses a simplified pore network (PN), and direct methods, which simulate flow using real porous media geometry.

Pore network modeling, an idea that was first applied in the 1950s^1^, is still widely used for porous media simulations, including single-phase transport modeling processes such as permeability estimations^2–4^. It can also be used to investigate multiphase transport models^2,5,6^, including modeling of the drainage-wetting hysteresis phenomenon^7^. PNM has also been used for reactive transport phenomena modeling including the dissolution-leaching phenomenon^8,9^ in the case of multiscale dual-porosity porous media^10^ and porous media advection-adsorption phenomena^11^.

The basis for PNM is a pore network representing the simplified pore geometry of a porous medium. Initial approaches to generating pore networks include statistical reconstruction and a grain-based approach^12,13^. In the case of statistical reconstruction, a pore network is created to meet a set of statistical characteristics, usually measured using intrusion porosimetry without including information about the real pore space geometry. Another approach, the grain-based algorithm, mimics the diagenesis of a porous media and simulates the deposition of grains forming the porous medium^14^. Currently, the possibility of direct observation of the pore space using X-ray computational tomography (CT) allows for the generation of pore network models using direct mapping algorithms, which can be divided into two main classes: medial axis^3,15^ and maximal ball^16,17^ algorithms. In addition to these approaches, alternative methods of pore network generation are available, particularly optimization techniques that can be employed for this purpose. Nejad Ebrahimi et al.^18^ proposed a genetic algorithm-based optimization method that focuses on minimizing the differences between CT images of the porous medium and the generated pore network. Hu et al.^13^ used a simulated annealing optimization for PN generation with a statistical reconstruction algorithm. Pore networks are substantial simplifications of pore space, and there were attempts to generate more realistic PN geometry, Raeini et al.^19^ proposed a modification of the maximal ball algorithm that generates a PN by taking into account non-spherical pore shapes by assigning additional volume to the pore corner regions. Other approach^20^ tries to use in the PN other than cylindrical, more natural in shape throat geometries. PN generation is a time consuming procedure needed substantial computational resources, recently new, more efficient method of PN generation, based on geometric domain decomposition was proposed^21^.

One alternative to PNM is a direct method of modeling the pore transport processes, which, unlike PNM, more accurately, exactly following pores’ shapes, represents the pore space. Computational fluid dynamics (CFD) modeling methodologies are used in this case, including the classical mesh-based Navier–Stokes (NS) modeling equation and Lattice Boltzmann (LB) method.

Complex flow geometries, like those of porous media, can be easily introduced with LB modeling. Due to the regular grids used in LB, native LB porous media is represented as a cubic pore surface approximation, which may impact simulation results^22^ due to the stair-step surface effect. An exact description of the pore space surface is also possible with LB, though it requires special treatment due to the boundary conditions^23^.

An alternative to LB modeling of transport in porous media is the NS CFD modeling technique. The finite volume method (FVM) for NS equations is one of the most frequently used differential equation discretization schemes utilized for porous media modeling because it is especially suitable for meshing complicated pore space geometry.

FVM has been used for single-phase transport modeling in porous media, including permeability estimation^24–26^. The FVM method is also capable of modeling multiphase transport phenomena at the pore scale^27–30^. Additionally, the volume of fluid (VOF) approach can be used for this purpose^31,32^ as well as for interface tracking. Non-Fickian^33^ behavior and reactive transport^34,35^ were also simulated.

FVM NS modeling may be coupled with other modeling techniques, such as the streamline method^36^ for chemical advection modeling, the LB method^37^ for coupled heat and water or water and chemical^38^ transport in porous media, and the direct element method (DEM) for fluid–solid interaction modeling^39^.

This paper aims to intercompare two methods used for saturated conductivity estimation: NS CFD and PNM. As the PNM relies on a highly simplified representation of the pore space, which may potentially impact simulation results, an attempt was made to quantify it. On the other hand, NS CFD technique rely on the numerical meshes resembling complicated pore-space geometry. The potential impact of the numerical mesh exactness with modelled pore medium geometry on the simulation results is also discussed. The error of estimation of the saturated conductivity using NS CFD approach was proposed. The comparison is based on a wide range of porous materials with a broad granulometric composition to achieve different pore space geometrical characteristics. Real porous media high-resolution CT images were used for these comparisons.

## Results and discussion

### Pore space porosity analysis

The saturated conductivity for all examined samples was estimated using three different modeling approaches. Thresholded images of analyzed pore space gathered from CT scans were the initial source of information for all of the intercompared modeling approaches. However, the information about pore space geometry was used differently by each of the modeling techniques. The NS modeling methodology considers the complete physical description of the transport phenomena and relies on true pore space geometry in the numerical mesh generation procedure. Meanwhile, pore network modeling relies on a simplified PN consisting of spherical pores and cylindrical throats.

**Figure 1:**
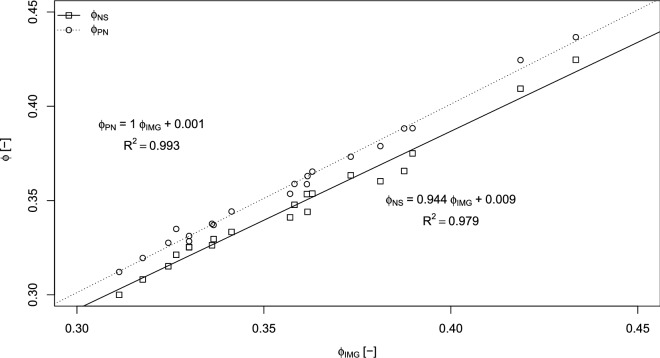
Total porosities of generated numerical meshes used for NS modeling (Φ_NS_) and pore networks (Φ_PN_) used for PN modeling as a function of the total porosity determined from CT imagery (Φ_IMG_) (figure created using RStudio 1.3 software https://rstudio.com/).

In the KC approach, pore space geometry was considered only in the form of two global measures: total porosity and specific surface (presented in Table 1). Specific surface and total porosity values for each of the pairs of samples, prepared from the same sand (Table 2), were similar, which indicates that the specimens used for CT scanning were successfully homogeneously prepared. The total porosity of the thresholded 3D CT image (Φ_IMG_) used for the NS mesh and PN network generation was treated as a reference for comparisons with the total porosities of the NS meshes, Φ_NS_, and PN networks, Φ_PN_, which should be the same—Fig. 1 presents this dependence. Pore network porosity, Φ_PN_, has approximately the same values as image porosity, Φ_IMG_. As can be seen in Fig. 1, Φ_PN_ followed almost a 1:1 relationship with Φ_IMG_. We may conclude that the PN network generation procedure worked well in this context.

**Table 1:** Pore space geometry, numerical mesh, and pore network stats of samples.

Name of sample	Numerical mesh stats			Pore networks stats			3D pore space image stats		NS mesh generation time (h)	PN network generation time (h)	Tortuosity (–)	Ksat KC (m s^−1^)	Ksat PN (m s^−1^)	Ksat NS (absolute error/relative error) (m s^−1^)	Ksat NS relative error (%)
	Number of cells (–)	Number of deleted cells (–)	Ratio of deleted cells (–)	Number of pores (–)	Number of throats (–)	Median throat length to radius ratio (–)	Specific surface (m^−1^)	Total porosity (m^3^ m^−3^)							
1d	23,203,800	741	3.19E−05	16,875	33,356	22.6709	12,950	0.33	5.3	18.3	1.418	1.17E−03	7.87E−04	5.27E−04 (2.52E−05)	4.8
1u	23,258,318	3392	1.46E−04	10,645	20,327	23.5124	13,080	0.33	15.6	19.3	1.443	1.10E−03	6.87E−04	4.87E−04 (2.61E−05)	5.4
2d	31,587,606	1452	4.60E−05	15,510	44,658	20.0673	19,070	0.34	20.6	27.4	1.493	5.83E−04	3.20E−04	2.32E−04 (1.60E−05)	6.9
2u	31,574,657	1346	4.26E−05	14,649	44,579	19.549	19,000	0.34	8.1	23.5	1.501	5.84E−04	3.24E−04	2.23E−04 (2.18E−05)	9.8
3d	51,814,954	3318	6.40E−05	41,435	143,724	18.8694	29,860	0.39	30.3	28.9	1.588	4.26E−04	1.76E−04	1.38E−04 (2.73E−05)	19.8
3u	52,118,200	2848	5.46E−05	38,742	146,382	17.1822	29,610	0.38	24.8	30.2	1.574	4.04E−04	1.56E−04	1.28E−04 (2.45E−05)	19.1
4d	85,459,910	2296	2.69E−05	379,328	2,140,324	12.6915	60,490	0.42	23.8	16.6	1.644	1.45E−04	1.60E−05	4.21E−05 (3.26E−06)	7.7
4u	86,628,845	6898	7.96E−05	204,628	1,160,151	12.1469	53,420	0.43	20.6	16.3	1.538	2.18E−04	3.01E−05	7.34E−05 (5.20E−06)	7.1
5d	46,720,483	10,370	2.22E−04	56,163	229,266	15.6947	31,560	0.31	46.9	9.8	1.659	1.56E−04	3.99E−05	6.91E−05 (8.34E−06)	12.1
5u	44,983,922	5317	1.18E−04	37,787	150,473	15.9696	26,460	0.32	49.1	14.1	1.621	2.41E−04	9.73E−05	1.02E−04 (1.01E−05)	9.9
6d	33,270,506	3179	9.56E−05	13,562	40,799	21.819	19,410	0.33	26.9	15.7	1.537	5.20E−04	2.76E−04	2.08E−04 (9.72E−06)	4.7
6u	33,417,727	2146	6.42E−05	16,416	49,038	23.0021	19,550	0.32	9.6	15.5	1.521	4.79E−04	2.57E−04	2.06E−04 (1.96E−05)	9.5
7d	59,107,329	14,250	2.41E−04	70,348	349,676	13.2417	34,670	0.37	55.8	11.7	1.627	2.70E−04	4.93E−05	9.92E−05 (8.86E−06)	8.9
7u	62,195,520	14,841	2.39E−04	104,741	520,133	13.1857	37,830	0.36	51.0	13.2	1.684	1.88E−04	3.14E−05	7.03E−05 (1.07E−05)	15.2
8d	70,414,495	15,692	2.23E−04	152,095	784,536	13.1265	45,140	0.39	14.6	18.1	1.686	1.91E−04	4.71E−05	7.07E−05 (9.25E−06)	13.1
8u	66,639,873	18,813	2.82E−04	129,556	638,073	12.9524	44,460	0.36	11.6	10.1	1.766	1.44E−04	2.89E−05	5.32E−05 (8.93E−06)	16.8
9d	37,131,330	5098	1.37E−04	14,620	56,674	16.6269	22,030	0.36	6.4	14.3	1.508	5.94E−04	2.67E−04	2.53E−04 (2.19E−05)	8.7
9u	43,191,834	5889	1.36E−04	24,846	105,354	14.8311	25,980	0.34	8.0	12.8	1.581	3.32E−04	1.27E−04	1.39E−04 (1.09E−05)	7.8
10d	60,180,476	11,233	1.87E−04	68,597	341,501	12.4603	33,660	0.36	10.7	12.6	1.619	2.50E−04	5.37E−05	9.46E−05 (7.10E−06)	7.5
10u	58,963,333	11,378	1.93E−04	72,263	353,914	12.6015	35,910	0.36	11.6	13.6	1.609	2.12E−04	5.98E−05	8.19E−05 (8.05E−06)	9.8

**Table 2:** Milled sand fraction mixing scheme.

Milling time (min)	Proportion of each milled fraction in prepared samples (%)						
	s4	s5	s6	s7	s8	s9	s10
5	0	0	100	50	0	50	33.3
10	0	100	0	0	50	50	33.3
20	100	0	0	50	50	0	33.3

In contrast, a comparison between Φ_NS_ and Φ_IMG_ reveals systematic underestimation. The average value of the differences between Φ_IMG_ and Φ_NS_ was 0.01 [m^3^ m^−3^]. The total porosity of NS meshes was underestimated by 1.5% to 5.8%. Such differences in the total porosities of the numerical meshes used for these simulations and total porosities of pore space images cannot be simply neglected, as they could potentially impact the calculation results.

To deal with this issue, the error of saturated conductivity determination using the NS approach was estimated using proposed error estimation approach (Eq. 11). This approach assumes that the NS modeling error is related to the decrease in total porosity of a pore space following the same dependence as in the KC modeling approach. The absolute and relative values of these errors are presented together with estimated K_sat_ values in Table 1. The error due to numerical mesh total porosity underestimation introduced via K_sat_ estimation using the NS approach was in the range of 4.6–19% of the estimated K_sat_ value. The error was on the average 10% for analyzed samples. The relative error seem to be higher for fine grained samples. The lowest values of the relative error was achieved for samples prepared from coarsest sand material.

### Technical aspects of simulation and modeling

Table 1 also provides information about the generated numerical meshes and pore networks. The generation of the NS meshes and PN networks was the most computationally demanding task in this study. A multicore workstation with 512 GB RAM was used for this purpose. The size of the RAM was a limiting parameter that prevented the generation of more detailed NS meshes. The NS meshes that were generated consisted of 20 million cells in the case of the simplest pore space geometries and up to almost 90 million cells in the case of the samples with the highest specific surface. The files containing NS meshes have sizes ranging from ~ 3.5 to 11.5 GB. NS mesh generation using parallel mesh generation software took anywhere from five to 55 h. Pore network generation took a similarly long time, from 10 to 30 h each. Pore network generation time was substantially shortened from initial runs by parallelization of the initial ball searching step of the original^17^ pore network generation code. Compared to the NS mesh and PN network generation times, saturated conductivity simulations were computationally feasible tasks. The difference between PN and NS computation times was significant; PN simulations took minutes while NS simulations required approximately one hour each.

During the automated procedure of mesh creation based on pore space geometry, despite quality constraints enforced by the mesh creation utility, some of the cells were of bad quality. These cells frequently displayed a large skewness that could impair the stability and quality of the calculations. As a final step in the mesh creation procedure, these bad cells were simply removed from the mesh. The number of cells removed from the NS meshes due to their failure to meet quality constraints is also presented in Table 1. Generally, the number of cells removed was minuscule and could be safely neglected. It is worth mentioning that removing low-quality cells had nothing to do with the NS mesh total porosity underestimation. On average, 0.015% of cells were removed from each NS mesh, while the level of total average porosity underestimation was 3.6%.

The total porosity underestimation for NS meshes could be attributed to several different sources. First, due to the constraints imposed on the size of the smallest possible cell size, the mesh generation utility could not generate a mesh in narrow regions where sand grains touch or are close to each other. Fortunately, these narrow regions should not greatly impact fluid flow, as in the case of a full saturation flow, it is a known phenomenon that the main flowpaths to transport dominate contributions to the overall pore space flow. Second, a possible cause of total porosity underestimation is the impossibility of mesh generation in the detached areas of a pore space. However, in this case, this should not be a problem, as these regions naturally do not take fluid flow into account.

**Figure 2:**
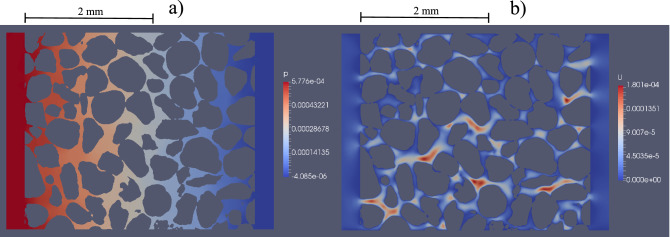
Simulated water transport through sample 1d shown at a central cross-section of the sample: **(a)** kinematic pressure field p [m^2^ s^−2^], **(b)** velocity magnitude field U [m s^−1^] (figure created using Paraview 5.3 software https://www.paraview.org/).

When the numerical mesh was generated, NS equations together with boundary conditions allowed for the determination of the pressure and velocity fields in the simulated porous media flow. Figure 2 presents an example of the longitudinal cross-section along the direction of water flow in sample 1d. The uniformly changing pressure can be observed in Fig. 2a and has a pressure gradient enforced by the NS model boundary conditions. The velocity magnitude field is presented in Fig. 2b, in which areas of higher velocity, caused by the narrow pores, may be observed.

### Saturated conductivity—the dependence on the modeling approach used for estimation

The NS modeling methodology, unlike PN modeling, accounts for full physical descriptions of the transport phenomena. Additionally, NS modeling relies on true pore space geometry and is presented here as a reference.

**Figure 3:**
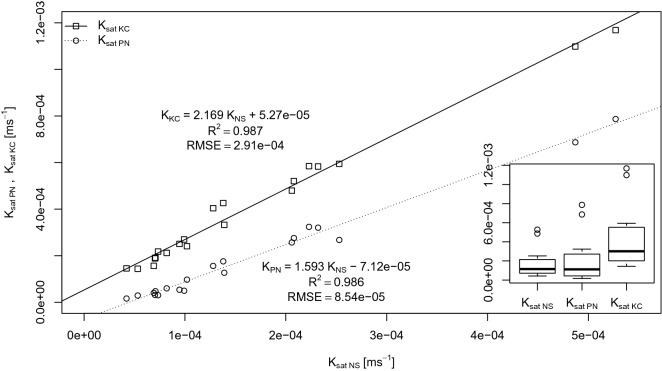
Main plot—saturated conductivities estimated via pore network modeling (K_sat PN_) and Kozeny–Carman (K_sat KC_) modeling compared with the Navier–Stokes (K_sat NS_) estimations. The subplot presents boxplots of the K_sat PN_, K_sat KC,_ and K_sat NS_ datasets (figure created using RStudio 1.3 software https://rstudio.com/).

Other modeling approaches (e.g., PN and KC) were compared with the NS model. The results of this comparison are shown in Fig. 3 together with linear models linking saturated conductivities determined by the PN and KC methodologies with their NS-based counterparts. Based on these results one can conclude that, compared to the NS model, the KC-based modeling approach systematically overestimates saturated conductivity values, as the slope parameter of the linear model (LM) is greater than one (2.169) and the intercept is positive. The average ratio of K_satKC_ to K_satNS_ (K_satKC/NS_) is even larger than the slope parameter and is equal to 2.624. On average, samples analyzed using the KC equation-based estimations are almost three times greater than NS-based K_sat_ estimations. The difference between K_satNS_ and K_satKC_ can also be observed in the subplots of the boxplots presented in Fig. 3.

The KC equation is known to be only a rough estimation^40^ of conductivity, though some discrepancy is not unexpected. Different studies have shown that KC underestimates^40,41^ or overestimates^42^ saturated conductivity depending on the porous medium for which the KC equation is used. In the case of Kuang et al.^40^, the authors used a tortuosity estimation method based on CT porous media images, which generated high tortuosity values of ~ 4.5 that could lead to conductivity underestimation. Our results, as they relate to the KC saturated conductivity estimation, are not as overestimated as those reported by Mostaghimi et al.^42^, in which KC estimation was also compared with NS estimation. This difference might be due to the different pore space characteristics of the porous material studied. The greatest overestimation was reported by Mostaghimi et al.^42^ for samples with porosities lower than 0.25, which were not observed in the samples used in this study. Taheri et al.^43^ reported high levels of agreement between KC and NS saturated conductivity estimations, but their work was related to a virtual homogeneous sand porous medium built from spherical particles. This medium very closely resembled the KC equation assumptions. The accuracy of KC estimations may be problematic, but considering how easily this estimation can be performed in comparison to the application of NS and PN models, the KC method is suitable in some situations.

Estimations of saturated conductivity via PN modeling are also presented in Fig. 3. In the case of PN estimations, the LM model slope parameter is equal to 1.593, but the intercept is negative. When the average ratio of the K_satPN_ to K_satNS_ (K_satPN/NS_) is calculated, it equals 0.927. This result shows that PN-based estimation of the saturated conductivity gives, on average, practically the same results as NS-based estimations. Boxplots (Fig. 3) confirm that these two sets (i.e., K_satPN_ and K_satNS_) are comparable. This conclusion is also confirmed by a two-sided paired Wilcoxon signed-rank test applied to the saturated conductivity estimation data. In the case of the comparison between the NS- and PN-based estimations, the result of the test (p-value) is equal to 0.3884, confirming the hypothesis that the medians of both sets are equal. In the case of the comparison between the NS- and KC-based estimations, the p-value, 1.9e-6, is far less than the significance level α = 0.05; thus, the medians of these sets are not equal, which confirms previous statements. Similarly, high levels of agreement between PN and NS estimations were found by Mehmani and Tchelepi^44^ for artificial 2D porous media micromodels. A similar comparison to the 3D porous media was performed, but for the virtual porous media; Yang et al.^26^ analyzed one artificial sphere pack porous medium and found high agreement between PN- and NS-estimated saturated conductivity. Finally, real 3D porous media were also analyzed by Jiang et al.^3^ which demonstrated similarly good agreement when three low resolution (256^3^ voxels @ 6 µm) rock samples were studied. Our study, which includes larger and higher-resolution real porous media geometry (~ 1400^3^ voxels @ 2 µm), confirmed the equivalence of NS and PN estimations.

**Figure 4:**
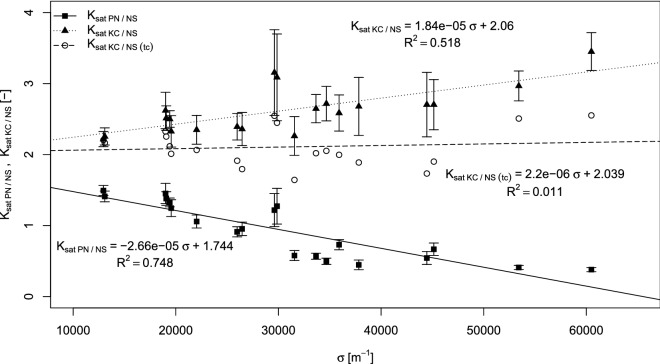
Relationship between the ratios of saturated conductivities determined by the pore network (K_sat PN/NS_) and Kozeny–Carman (K_sat KC/NS_) to saturated conductivities estimated by Navier–Stokes modeling (figure created using RStudio 1.3 software https://rstudio.com/).

The relationship of NS-determined saturated conductivity to its PN and KC counterparts, expressed by the average of the aforementioned ratios (i.e. K_satKC/NS_ and K_satPN/NS_), does not completely describe the situation. Regression analysis demonstrates the dependence of both properties, K_satKC/NS_ (r = 0.72) and K_satPN/NS_ (r = -0.86), on the sample’s specific surface, determined based on thresholded images—the same images which were used for the PN network and NS mesh generation. This dependence is presented in Fig. 4, where K_satKC/NS_ falls within the range of 2.2–3.5 and K_satPN/NS_ is in the range of 0.4–1.5.

It is important to note that if K_satKC/NS_ increases, but K_satPN/NS_ decreases with the increase of the specific surface σ [m^-1^]. This means that these opposite trends in the ratios are not caused by the dependence between K_satNS_ and σ, as if that were the case, the trends would follow a similar pattern. To ensure the possible influence of the K_satNS_ estimation error on the values of K_satKC/NS_ and K_satPN/NS_, their errors (presented as error-bars in Fig. 4) were calculated based on error propagation theory. Since the increasing specific surface is related to the particle size distribution of the material from which the samples were prepared, which in turn influences pore space complexity, we hypothesized that the observed trend of K_satKC/NS_ might be connected to the changing tortuosity of the pore space.

To validate this hypothesis, the tortuosities of the samples were estimated (see Table 1) based on the streamlines analysis. The lowest values of tortuosity for the bed of spherical grains, close to the theoretical value of 1.414, were estimated for samples 1u and 1d, which were made of the coarser fraction of sieved sand grains. However, the majority of samples were made of the milled sand, which consisted mostly of non-spherical grains where one might expect higher tortuosity to occur. As a result, tortuosity is positively correlated (r = 0.71) with the image specific surface and the highest values of tortuosity ~ 1.6–1.7 were achieved in samples with a higher fraction of finely-milled sand.

When these tortuosity estimations were used in the KC Eq. (4) instead of the constant value 1.414, the KC estimations no longer depended on the value of σ (see data series K_satKC/NS (tc)_ in Fig. 4). This confirms the hypothesis about tortuosity dependence on the pore space complexity.

**Figure 5:**
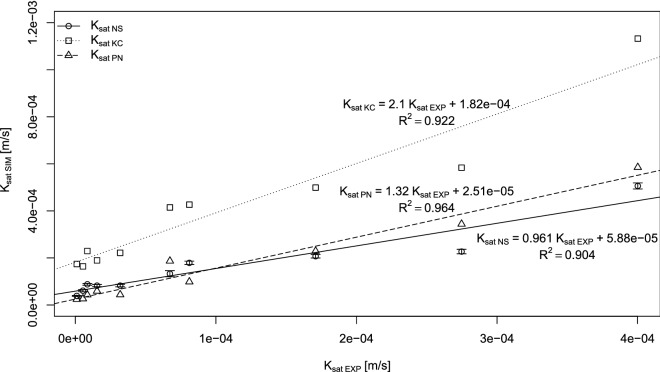
Comparison between measured and simulated water conductivities (K_satEXP_—experimentally determined values for specimens, K_satSIM_—values determined based on: NS, PN, and KC estimations; figure created using RStudio 1.3 software https://rstudio.com/).

The results obtained from numerical estimations and from Kozeny-Carman approach were also subjected to experimental verification (Fig. 5). As per one specimen, for which K_sat_ was measured, two samples belongs simulated and measured values could not be compared directly. Saturated conductivities estimated for the samples were recalculated to the specimen’s values according to Eq. (2). In case of NS estimations, error using proposed approach (Eq. 11) was also for all samples determined. On the figure (Fig. 5) for all specimens K_satNS_ errors are presented—they were recalculated based on error propagation theory from appropriate samples’ error estimates.

## Conclusions

The PN-estimated saturated conductivity (K_satPN_) was found to be statistically equivalent to NS-determined saturated conductivity values (K_satNS_). The average value of the K_satPN_/K_satNS_ ratio was equal to 0.927.

The KC equation-based estimation (K_satKC_) overestimated saturated conductivity by more than double (2.624) the K_satNS_ estimate. However, despite high levels of overestimation, both sets of values are well-correlated with R^2^ = 0.99.

The total porosities of the generated PN were the same as the total porosities of images used for their creation. Total porosities of numerical meshes were on average lower by 0.01 [m^3^ m^−3^] than the porosities of the corresponding reference images.

Proposed error estimation approach was used for relative error determination of the NS saturated conductivity modelling. The error was on the average 10% for analyzed samples. The minimum value of the error was 4.6% and maximum 19%. The relative error seem to be higher for fine grained samples. The lowest values of the relative error was achieved for samples prepared from coarsest sand material.

The tortuosity of 20 samples was estimated. The value of the tortuosity determined for the samples prepared from coarser fractions of sand approximated the theoretical tortuosity value of the porous medium built from spherical grains, 1.4. The tortuosities of samples prepared from milled sand (non-spherical grain material) were higher. The value of tortuosity was correlated (r = 0.71) with the porous medium-specific surface, reaching the highest values of 1.6–1.7 for samples prepared from the finest sand material.

## Materials and methods

### Specimens preparation

Specimens were prepared by mixing different unimpaired sieved and milled sand fractions. The first step in specimen preparation was to sieve the sand using a 0.5 mm sieve. This raw sand was then milled in the planetary mill (Pulverisette 6 classic line, Fritsch, Germany, Idar-Oberstein). The raw sand was milled for 5, 10, and 20 min to achieve milled sand fractions with different particle size distributions.

**Figure 6:**
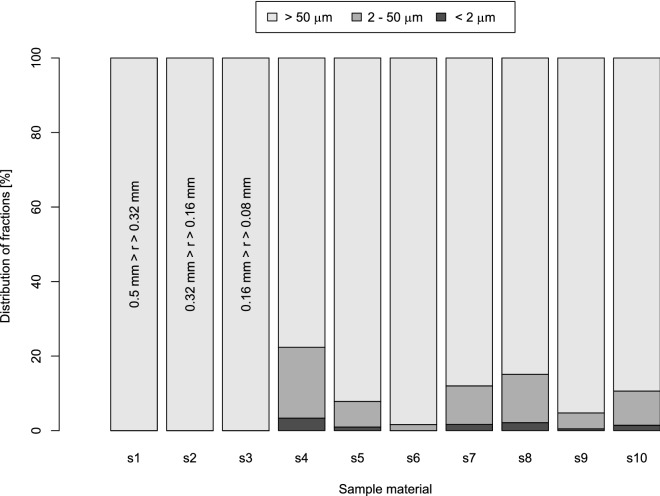
Particle size distribution of sand material used for specimen preparation (figure created using RStudio 1.3 software https://rstudio.com/).

10 specimens were then prepared from a mixture of sieved sand and milled sand fractions. Specimens s1, s2, and s3 were prepared by sieving raw sand through 0.32, 0.16, and 0.08 mm sieves, respectively. Table 2 presents the mixing scheme used to create material with diverse particle size distributions (PSD) for the other specimens. Each prepared specimen’s material prior to the specimen preparation was measured using a laser diffractometry method (LDM) to verify the PSD that was achieved (Fig. 6). A Malvern Mastersizer 2000 with a measurement range of 0.02 µm to 2 mm was used for PSD determination. To obtain homogeneity in the sand suspension, a Hydro G dispersion unit was used, with a pump speed of 1750 rpm and stirrer speed of 700 rpm. Light intensity, measured with the detectors, was recalculated into PSD according to the Mie theory (ISO 13,320:2009, 2009). The Mie model parameters include an absorption coefficient of 0.1 and a refractive coefficient of 1.52^45^. Due to this specimen preparation procedure, it was possible to evaluate the potential impact of the pore space geometry on saturated conductivity modeling.

**Figure 7:**
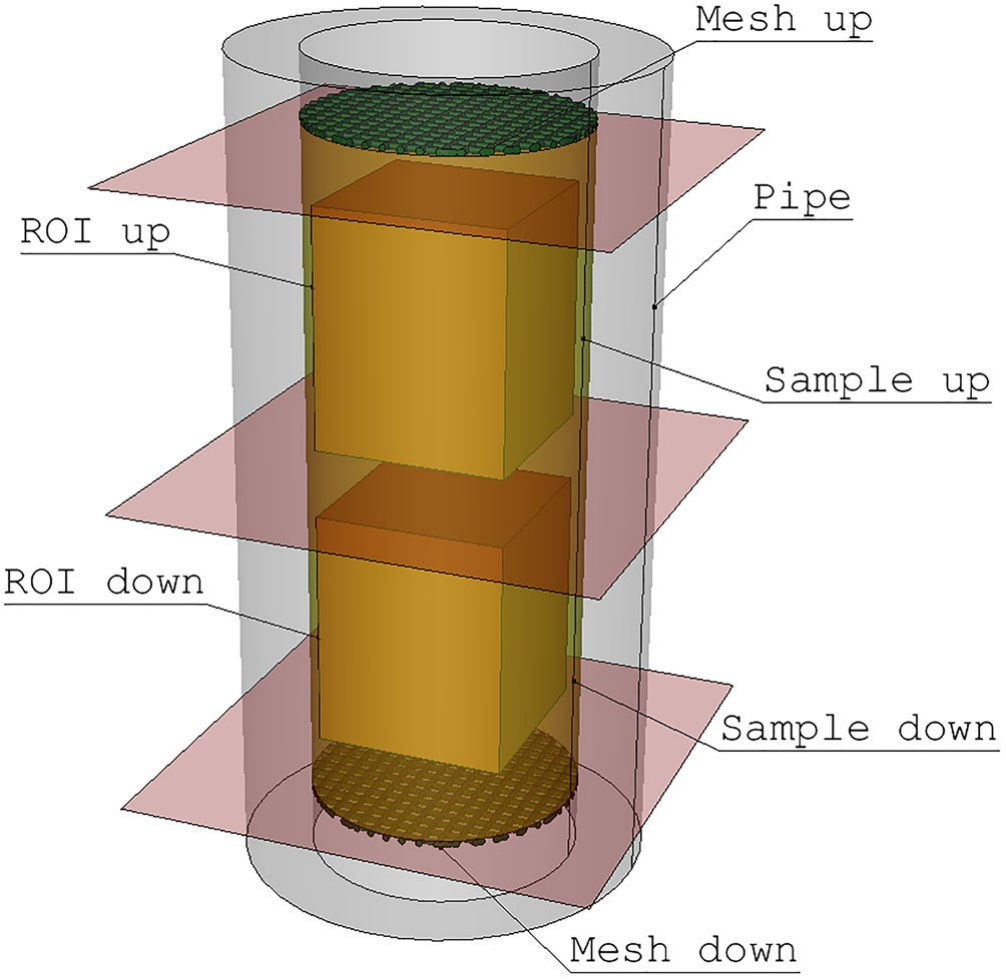
The geometry of the specimen (ROI up—the region representing upper sample, ROI down—region representing lower sample; Figure created using Salome 9.6 software https://www.salome-platform.org/).

The specimen was prepared in a polypropylene tube (4 mm internal diameter) of low X-ray absorbance (Fig. 7). An adequate amount of the specimen’s material was poured into the cylinder to achieve a 10 mm stack inside the container. The specimen was subsequently compacted in the pipe by vibration. At the bottom and top of the specimen’s material stack bronze meshes were placed to keep the material in place. In the case of the specimens (s4, s5, s6, s7, s8, s9, and s10) additionally, the circle cut from filter paper was placed between the mesh and the material to avoid loss of the fine particles from the specimen. Bronze meshes were used as they were stiff enough to support properly the specimen’s material, but there was a drawback of this choice. As the metal is far less penetrated by X-rays than sand, strong streaking artifacts are observed in the reconstructed scans near the mesh. As a result, the pore space in the neighborhood of the bronze meshes can’t be observed. That was also a reason for the preparation of relatively high (10 mm) specimens as we would like to ensure a big enough part of the specimen could be CT analyzed to ensure the reasonable comparison between modeling and measurement of the saturated conductivity. As the height of the area which is examined using CT is roughly equal to its diameter, in the 4 mm wide and 10 mm high specimen two samples area were defined.

### X-ray CT sample analysis

An X-ray CT scanner (GE Nanotom 180S) was used to examine the specimens with a 180 kV/15 W microfocus X-ray tube. For each specimen, two scans were made, one in the upper part of the specimen and the second in the lower part (Fig. 7). Two regions from each specimen were scanned to achieve the highest possible CT resolution, which limited the dimensions of the scanned object. This also increased the number of analyzed samples. As a result, 20 scans were made. They will be referred to as “10d” and “10u,” for example, where “10d” identifies the lower (downward) part of the 10th specimen and “10u” is the upper part of the 10^th^ specimen.

For each scan, a series of 1200 2D radiograms were collected, with the specimen rotating through the full angular range using a rotation step of 0.3°. Each 2D radiogram was averaged over 15 images to reduce noise. The images were recorded using a detector with a resolution of 2284 × 2304 pixels registering images at the 14-bit gray-level depth. The X-ray source operated at 90 kV with a 120 μA cathode current and tungsten exit window. Immediately before each scan, a short pre-scan was performed, which lasted for 30 min to preheat the specimen and minimize the impact of thermal expansion during the primary scan for three-dimensional (3D) image reconstruction. The voxel size achieved in these CT scans was 2 µm.

### 3D image reconstruction and processing

**Figure 8:**
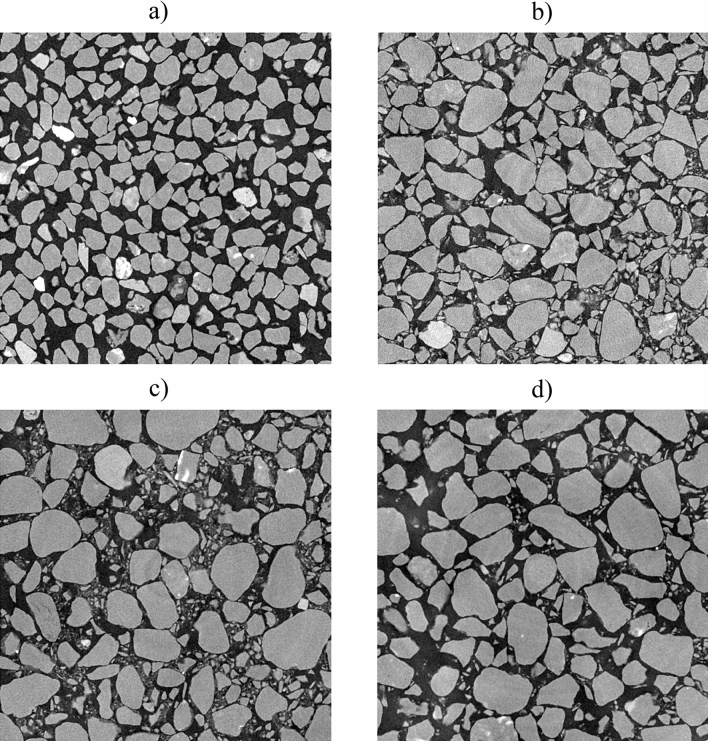
Cross-sections of the selected ROIs of the samples: **(a)** S3u, **(b)** S5u, **(c)** S7u, and **(d)** S9u (figure created using Fiji 1.5 software https://fiji.sc/).

Tomographic reconstruction was performed using a series of radiograms (DatosX version 2.0.1, General Electric). The beam hardening correction was not necessary, although the exit window filter was not used during the X-ray scan. From the obtained 3D volume representing the sample and a part of the tube, a cubic region of interest (ROI) of 1400 × 1400 × 1700 voxels was chosen for further processing. Cross-sections of the ROIs of selected samples are presented on the Fig. 8. A median filter with a three-pixel kernel diameter was applied to the ROI. The next step was thresholding using an IsoData thresholding method^46^. Image processing was conducted with Fiji and VGStudio MAX 2.1 (Volume Graphics). The thresholded images serve as a common starting point for both the PN and NS modeling approaches.

### Measurement of the specimen’s saturated conductivity

**Figure 9:**
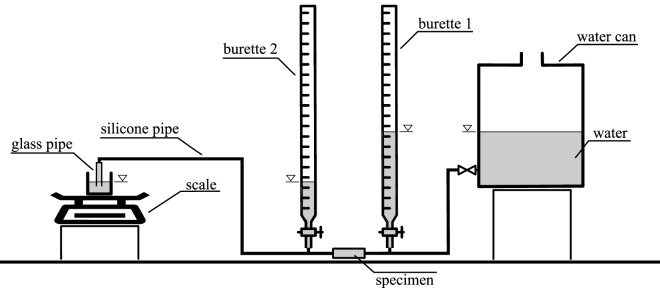
The schema of the saturated conductivity measurement laboratory setup (figure created using Inkscape 0.99 software https://inkscape.org/).

For each of ten prepared specimens, saturated water conductivity was measured. The measurement was based on the static pressure head principle (Fig. 9). The pressure difference was fixed, and water flux was measured, then saturated conductivity K [m s^−1^] was calculated using Darcy's law:

1 $$K=\frac{\rho gq\Delta p }{\mathrm{\Delta l}}$$

where *q* [m s^−1^] is fluid flux, *Δp* is the pressure difference [Pa] along the sample length *Δl* [m], *g* is the gravitational constant [m s^−2^], and *ρ* is fluid density [kg m^−3^].

The pressure difference was adjusted by changing the water level in the water tank. The value of the difference in the pressure head was read from the water level difference in the two burettes mounted before and after the specimen. Water flux was measured based on the scale readings on which the container collecting the water flowing through the specimen was placed.

The idea of the measurement is simple, but due to very low water fluxes—especially for fine-grained specimens—some additional factors had to been taken into account. To avoid the capillary forces' influence on the scale reading, the water container located on the scale was initially filled up to the level little higher than the end of the glass pipe placed in it. Some of the measurements were long enough to force the water weight loss correction due to water evaporation from the container placed on the scale. The evaporation rate from the container in the constant air temperature kept in the laboratory was determined upfront. The last factor that needed correction was the influence of the bronze meshes and paper filter placed at the ends of the specimen’s material. Five empty (consisting only from meshes and filter paper) specimens were prepared, and dependence between water flux and pressure head difference was determined for them. Based on averaged results calibrating function linking flux with pressure head difference was prepared. Then during regular measurements, based on registered water flux, appropriate pressure head difference was subtracted from the value read from the burettes.

The results of measured specimen’s saturated conductivities were compared with values of samples conductivities estimated using different methods. There values cannot be compared directly. But taking into account that two subsequent samples are defined in one specimen (Fig. 7) one may calculate effective water conductivity K_eff_ of two stacked samples with conductivities K_1_ and K_2_, as a harmonic average:

2 $$\frac{1}{{K}_{eff}}=\frac{1}{{K}_{1}}+\frac{1}{{K}_{2}}$$

### Pore network saturated conductivity modeling

**Figure 10:**
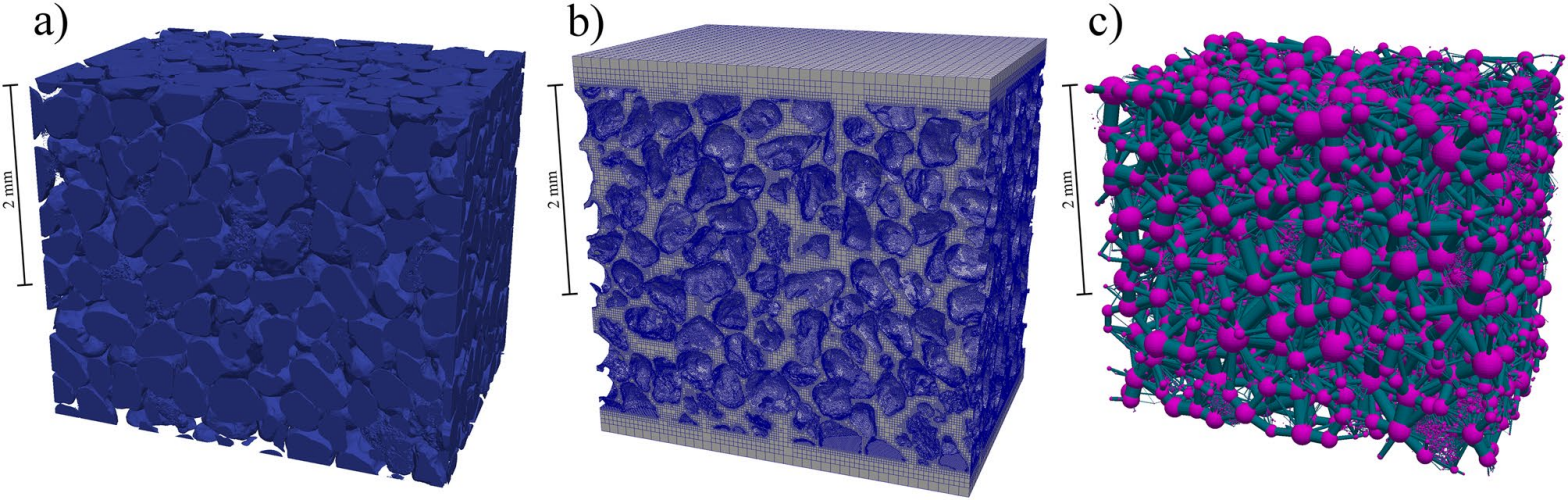
Different approaches to pore space representation: **(a)** triangulated pore space-solid boundary, the result of XRT scan, **(b)** numerical mesh spreading pore network, aligned to real pore space geometry, and c) simplified pore network representation by the pore network model. All three visualizations are based on sample 1d (figure created using Paraview 5.3 software https://www.paraview.org/).

The thresholded 3D images were processed using the network extraction code after applying a maximal ball algorithm^17^. The result was a dataset containing information about the spherical pores and cylindrical throats including dimensions, spatial coordinates, and connections^47^. A simplified pore network (Fig. 10c) was then used for permeability calculations based on the assumption of laminar flow, the Hagen-Poiseuille formula validity for flow in the throats, and mass conservation for each pore-throat connection^48^. The boundary condition pressure difference was set between the PN input and output nodes, resulting in fluid flow throughout the network.

### Navier–Stokes saturated conductivity modeling

The NS FVM approach is used to model a single-phase, laminar, steady-state flow of incompressible fluid through a porous medium. The set of equations used for the model contains the following momentum balance equation:

3 $$\frac{D}{Dt}\left(\rho \stackrel{-}{u}\right)=-\nabla \cdot \widehat{T}-\nabla {p}_{d}+\stackrel{-}{F}$$

where *ρ* is the local fluid density [kg m^−3^], $\stackrel{-}{u}$ is fluid velocity [m s^−1^], ${p}_{d}$ is pressure [Pa], $\widehat{T}$ is the momentum tensor [N m^−2^], and $\stackrel{-}{\mathrm{F}}$ is the external forces vector [N m^−3^].

Additionally, a mass balance for the equation was considered:

4 $$\nabla \cdot \stackrel{-}{u}=0$$

To solve the NS equations, simpleFoam software was used, which is a part of the OpenFOAM CFD toolbox. The solver implements a semi-implicit method for pressure-linked equations (SIMPLE) algorithm to solve the momentum balance equation.

Appropriate boundary conditions were established in the simulation. The fluid velocity was fixed to 10^–5^ m s^−1^, and the pressure was fixed to 0 Pa on the input patch and zero gradient Neuman conditions on the output patch. Input and output patches were equivalent to the top and bottom sides of the sample. No-slip boundary conditions were applied to the pore walls. The saturated conductivity was calculated from Darcy's law using the pressure drop and fluid velocity at the input boundary.

The first step of an NS simulation using FVM is mesh preparation. The surface of the solid fraction was determined from the thresholded 3D images and approximated using a triangulated surface mesh (Fig. 10a), which was saved in an STL format. For pore space surface triangulation, Fiji software was used along with an isosurface module from the BoneJ plugin^49^. The mesh (Fig. 10b) was then created based on the STL file using the snappyHexMesh utility (a part of the OpenFOAM toolkit). Next, the generated mesh was checked for highly skewed cells and other deficiencies. Cells that did not meet quality constraints, in particular those with skewness greater than four, were removed to avoid impairing the quality or numerical stability of the simulations. A very small number, not greater than 0.0282% of the total number of cells, had to be removed.

### Kozeny–Carman conductivity estimation

In addition to estimation via the physical modeling approach, the saturated conductivity of porous media can be also estimated using a phenomenologically derived formula, the KC equation (Eq. 4), which estimates saturated conductivity based on the total porosity and specific surface area of the porous medium. The equation is written as^40^:

5 $${K}_{KC}=\frac{{\phi }^{3}}{{{C}_{o}{\tau }^{2}(1-{\phi }^{2})\sigma }^{2}} \frac{\rho g}{\mu }$$

where *K*_*KC*_ is saturated conductivity [m/s], *Φ* is the total porosity [–], *σ* is the specific surface area (pore space surface area per sample volume) [m^−1^], *C*_*0*_ is the Kozeny constant, *τ* is the tortuosity [–], ρ is the fluid density [kg m^−3^], μ is the dynamic viscosity of the fluid [kg m^−1^·s^−1^], and g is the gravitational acceleration constant [m s^−2^]. Different values of C_0_ and *τ* may be used for conductivity estimation. In this study, we used the most commonly used values, which are valid for equally-sized spherical granular material: C_0_ = 2.0 and *τ* = 1.414. Total porosity and specific area were determined based on the thresholded 3D images of the samples using an image analysis approach. Total porosity was determined using the voxel counting method. The specific surface was determined based on the surface area of the triangulated solid-void interface approximation.

### Saturated conductivity estimation errors

Saturated conductivity estimation relies on the modeling of numerical meshes. In principle, numerical meshes that are generated based on images of a porous medium should match its overall geometric characteristics (e.g., total porosity). Any discrepancies may lead to errors in the saturated conductivity estimation^50^. To account for this, some method of saturated conductivity error estimation is needed. Ideally, error estimation should be based solely on numerical modeling methodology, but it is not possible. As a an error estimation method, following KC equation-based approach can be used, as it relates total porosity and specific surface to saturated conductivity. Proposed approach assumes correlation between K_NS_ and K_KC_. This correlation in case of samples analyzed here was 0.99. And of course validity of Kozeny-Carman estimation for pore media in question. Correlation between K_NS_ and K_KC_ means that the following dependence is assumed:

6 $${K}_{NS}(\phi )=A{K}_{KC}(\phi )$$

where A is some constant and explicitly dependence on mesh porosity $\phi$ was noted. Based on error estimation theory, the error ΔK_NS_ related to porosity underestimation $\Delta \phi$ will be calculated using following equation:

7 $$\Delta {K}_{NS}\left({\phi }_{0}\right)={\left|\frac{\partial {K}_{NS}\left(\phi \right)}{\partial \phi }\right|}_{{\phi }_{0}}\Delta \phi$$

But taking into account Eq. (6) following equation will be derived:

8 $$\Delta {K}_{NS}\left({\phi }_{0}\right)={A\left|\frac{\partial {K}_{KC}\left(\phi \right)}{\partial \phi }\right|}_{{\phi }_{0}}\Delta \phi$$

Based on Eq. (5), using the dependence of KC saturated conductivity estimation on total porosity, Eq. (8) become:

9 $$\Delta {K}_{NS}\left({\phi }_{0}\right)=A{\left|\frac{{\phi }^{2}}{{{C}_{o}{\tau }^{2}(1-{\phi }^{2})\sigma }^{2}} \left(3+\frac{2{\phi }^{2}}{1-{\phi }^{2}}\right)\frac{\rho g}{\mu }\right|}_{{\phi }_{0}}\Delta \phi$$

Which, using Eq. (5) again could be rewritten as:

10 $$\Delta {K}_{NS}\left({\phi }_{0}\right)={{AK}_{KC}\left({\phi }_{0}\right)\left|\frac{3-{\phi }^{2}}{\phi \left(1-{\phi }^{2}\right)}\right|}_{{\phi }_{0}}\Delta \phi$$

which finally, after taking into account Eq. (6), becomes the formula for relative K_NS_ error estimation:

11 $$\frac{\Delta {K}_{NS}}{{K}_{NS}}\left({\phi }_{0}\right)={\left|\frac{3-{\phi }^{2}}{\phi \left(1-{\phi }^{2}\right)}\right|}_{{\phi }_{0}}\Delta \phi$$

The relative and absolute errors estimated based on this formula are presented in Table 1.

### Tortuosity estimation

Porous medium tortuosity is one parameter involved in saturated conductivity estimation using the KC approach. Tortuosity is related to pore space geometry and pore space complexity. It is also defined as the ratio of the length of a path between two points to the distance between them.

The tortuosity of porous media can be estimated using either direct analysis of possible paths in 3D porous media images^51^ or analysis of a simulated velocity field in a pore space. The velocity field analysis approach may be used to deduce tortuosity based on the averaged velocity magnitude to longitudinal velocity component ratio^52^ or streamlines length and distance analysis^53^. The latter approach was used in this work.

**Figure 11:**
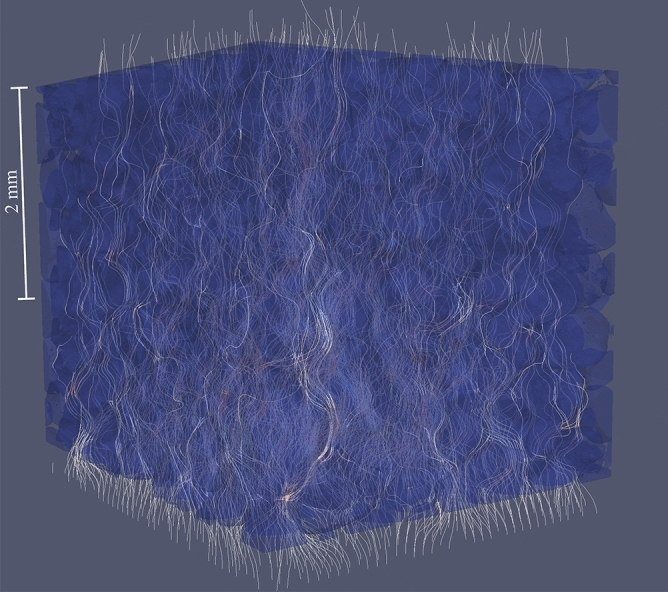
Schematic of streamlines analysis used for tortuosity estimation. Streamlines generated based on pore flow velocity field for the sample 1d (figure created using Paraview 5.3 software https://www.paraview.org/).

For all samples, fluid velocity fields were simulated using an NS modeling approach. Based on the information about pore geometry and the velocity field, streamlines were generated. For each sample, 1000 randomly distributed streamlines were seeded on the input patch, which were then propagated according to the velocity field (see Fig. 11). Finally, after discarding the parts of the streamlines that lay outside of the porous medium related mesh (i.e., in the areas next to input and output patches), the tortuosity value of the sample was estimated as the average of the tortuosities of the streamlines.

## References

[1] Fatt I (1956). The network model of porous media. Trans. AIME.

[2] Al-Kharusi, A. S. & Blunt, M. J. Multiphase flow predictions from carbonate pore space images using extracted network models. *Water Resour. Res.* **44**, W06S01 (2008).

[3] Jiang Z (2017). Pore network extraction for fractured porous media. Adv. Water Resour..

[4] Wang YD, Chung T, Armstrong RT, McClure JE, Mostaghimi P (2019). Computations of permeability of large rock images by dual grid domain decomposition. Adv. Water Resour..

[5] Aghaei A, Piri M (2015). Direct pore-to-core up-scaling of displacement processes: Dynamic pore network modeling and experimentation. J. Hydrol..

[6] Hannaoui R (2015). Pore-network modeling of trickle bed reactors: Pressure drop analysis. Chem. Eng. J..

[7] Rostami A, Habibagahi G, Ajdari M, Nikooee E (2015). Pore network investigation on hysteresis phenomena and influence of stress state on the SWRC. Int. J. Geomech..

[8] Köhne JM, Schlüter S, Vogel H-J (2011). Predicting solute transport in structured soil using pore network models. Vadose Zo. J..

[9] Mehmani A, Mehmani Y, Prodanović M, Balhoff M (2015). A forward analysis on the applicability of tracer breakthrough profiles in revealing the pore structure of tight gas sandstone and carbonate rocks. Water Resour. Res..

[10] de Vries ET, Raoof A, van Genuchten MT (2017). Multiscale modelling of dual-porosity porous media; a computational pore-scale study for flow and solute transport. Adv. Water Resour..

[11] Raoof A, Nick HM, Wolterbeek TKT, Spiers CJ (2012). Pore-scale modeling of reactive transport in wellbore cement under CO2 storage conditions. Int. J. Greenh. Gas Control.

[12] Xiong Q, Baychev TG, Jivkov AP (2016). Review of pore network modelling of porous media: Experimental characterisations, network constructions and applications to reactive transport. J. Contam. Hydrol..

[13] Hu, M.-C. *et al.* Development of Kriging-approximation simulated annealing optimization algorithm for parameters calibration of porous media flow model. *Stoch. Environ. Res. Risk Assess.* 1–12, 10.1007/s00477-018-01646-y (2019).

[14] Bryant S, Blunt M (1992). Prediction of relative permeability in simple porous media. Phys. Rev. A.

[15] Al-Raoush RI, Willson CS (2005). Extraction of physically realistic pore network properties from three-dimensional synchrotron X-ray microtomography images of unconsolidated porous media systems. J. Hydrol..

[16] Al-Kharusi AS, Blunt MJ (2007). Network extraction from sandstone and carbonate pore space images. J. Pet. Sci. Eng..

[17] Dong H, Blunt M (2009). Pore-network extraction from micro-computerized-tomography images. Phys. Rev. E.

[18] Nejad Ebrahimi A, Jamshidi S, Iglauer S, Boozarjomehry RB (2013). Genetic algorithm-based pore network extraction from micro-computed tomography images. Chem. Eng. Sci..

[19] Raeini AQ, Bijeljic B, Blunt MJ (2017). Generalized network modeling: Network extraction as a coarse-scale discretization of the void space of porous media. Phys. Rev. E.

[20] Zhao J, Qin F, Derome D, Kang Q, Carmeliet J (2020). Improved pore network models to simulate single-phase flow in porous media by coupling with lattice Boltzmann method. Adv. Water Resour..

[21] Khan ZA, Elkamel A, Gostick JT (2020). Efficient extraction of pore networks from massive tomograms via geometric domain decomposition. Adv. Water Resour..

[22] Yang X (2013). Direct numerical simulation of pore-scale flow in a bead pack: Comparison with magnetic resonance imaging observations. Adv. Water Resour..

[23] Moqtaderi, H. & Esfahanian, V. Evaluation of a new solid boundary implementation in the lattice Boltzmann method for porous media considering permeability and apparent slip. *Philos. Trans. R. Soc. A Math. Phys. Eng. Sci.* **369**, 2193–2201 (2011). 10.1098/rsta.2011.0095 21536565

[24] Trebotich D, Graves D (2015). An adaptive finite volume method for the incompressible Navier-Stokes equations in complex geometries. Commun. Appl. Math. Comput. Sci..

[25] Lesueur M, Casadiego MC, Veveakis M, Poulet T (2017). Modelling fluid-microstructure interaction on elasto-visco-plastic digital rocks. Geomech. Energy Environ..

[26] Yang X (2016). Intercomparison of 3D pore-scale flow and solute transport simulation methods. Adv. Water Resour..

[27] Raeini AQ, Bijeljic B, Blunt MJ (2015). Modelling capillary trapping using finite-volume simulation of two-phase flow directly on micro-CT images. Adv. Water Resour..

[28] Raeini AQ, Blunt MJ, Bijeljic B (2014). Direct simulations of two-phase flow on micro-CT images of porous media and upscaling of pore-scale forces. Adv. Water Resour..

[29] Verma R, Icardi M, Prodanović M (2018). Effect of wettability on two-phase quasi-static displacement: Validation of two pore scale modeling approaches. J. Contam. Hydrol. (article in press).

[30] Ferrari A, Jimenez-Martinez J, Borgne TL, Méheust Y, Lunati I (2015). Challenges in modeling unstable two-phase flow experiments in porous micromodels. Water Resour. Res..

[31] Rider WJ, Kothe DB (1998). Reconstructing volume tracking. J. Comput. Phys..

[32] Raeini AQ, Blunt MJ, Bijeljic B (2012). Modelling two-phase flow in porous media at the pore scale using the volume-of-fluid method. J. Comput. Phys..

[33] Bijeljic B, Raeini A, Mostaghimi P, Blunt MJ (2013). Predictions of non-Fickian solute transport in different classes of porous media using direct simulation on pore-scale images. Phys. Rev. E.

[34] Alhashmi Z, Blunt MJ, Bijeljic B (2015). Predictions of dynamic changes in reaction rates as a consequence of incomplete mixing using pore scale reactive transport modeling on images of porous media. J. Contam. Hydrol..

[35] Menke HP, Bijeljic B, Blunt MJ (2017). Dynamic reservoir-condition microtomography of reactive transport in complex carbonates: Effect of initial pore structure and initial brine pH. Geochim. Cosmochim. Acta.

[36] Pereira Nunes, J. P., Blunt, M. J. & Bijeljic, B. Pore-scale simulation of carbonate dissolution in micro-CT images. *J. Geophys. Res. Solid Earth* **121**, 558–576 (2016).

[37] Wu B, Xu Y, Zheng Y, Fan J (2013). A new cross-scaling method to deal with the porous flow problem. Theor. Appl. Mech. Lett..

[38] Liu M, Mostaghimi P (2017). High-resolution pore-scale simulation of dissolution in porous media. Chem. Eng. Sci..

[39] Han Y, Cundall PA (2013). LBM-DEM modeling of fluid - solid interaction in porous media. Int. J. Numer. Anal. Methods Geomech..

[40] Kuang X, Sansalone J, Ying G, Ranieri V (2011). Pore-structure models of hydraulic conductivity for permeable pavement. J. Hydrol..

[41] Latief FDE, Fauzi U (2012). Kozeny-Carman and empirical formula for the permeability of computer rock models. Int. J. Rock Mech. Min. Sci..

[42] Mostaghimi P, Blunt MJ, Bijeljic B (2013). Computations of absolute permeability on micro-CT images. Math. Geosci..

[43] Taheri S, Ghomeshi S, Kantzas A (2017). Permeability calculations in unconsolidated homogeneous sands. Powder Technol..

[44] Mehmani Y, Tchelepi HA (2017). Minimum requirements for predictive pore-network modeling of solute transport in micromodels. Adv. Water Resour..

[45] Bieganowski, A., Chojecki, T., Ryżak, M., Sochan, A. & Lamorski, K. Methodological aspects of fractal dimension estimation on the basis of particle size distribution. *Vadose Zo. J.* **12**, vzj2012.0064 (2013).

[46] Ridler TWW, Calvard S, Ridler TW, Calvard S, Ridler TWW, Calvard S (1978). Picture thresholding using an iterative selection method. IEEE Trans. Syst. Man. Cybern..

[47] Sochi T (2010). Non-Newtonian flow in porous media. Polymer (Guildf)..

[48] Valvatne PH, Piri M, Lopez X, Blunt MJ (2005). Predictive pore-scale modeling of single and multiphase flow. Transp. Porous Media.

[49] Doube M (2010). BoneJ: Free and extensible bone image analysis in ImageJ. Bone.

[50] Gackiewicz B, Lamorski K, Sławiński C (2019). Saturated water conductivity estimation based on X-ray CT images—Evaluation of the impact of thresholding errors. Int. Agrophys..

[51] Sansalone J, Kuang X, Ranieri V (2008). Permeable pavement as a hydraulic and filtration interface for urban drainage. J. Irrig. Drain. Eng..

[52] Ghassemi A, Pak A (2011). Pore scale study of permeability and tortuosity for flow through particulate media using lattice Boltzmann method. Int. J. Numer. Anal. Methods Geomech..

[53] Duda A, Koza Z, Matyka M (2011). Hydraulic tortuosity in arbitrary porous media flow. Phys. Rev. E.

